# Environmental Corporate Social Responsibility and Workplace Pro-Environmental Behaviors: Person-Organization Fit and Organizational Identification’s Sequential Mediation

**DOI:** 10.3390/ijerph191610355

**Published:** 2022-08-19

**Authors:** Ana Patrícia Duarte, Carla Mouro

**Affiliations:** 1 Business Research Unit, Iscte Instituto Universitário de Lisboa, 1649-026 Lisboa, Portugal; 2 Centro de Investigação e Intervenção Social, Iscte Instituto Universitário de Lisboa, 1649-026 Lisboa, Portugal; carla.mouro@iscte-iul.pt

**Keywords:** environmental corporate social responsibility, person-organization fit, organizational identification, pro-environmental behavior, sequential mediation model

## Abstract

Organizations’ environmental performance has come increasingly under scrutiny given the need for sustainable, low-carbon economies. Workers’ pro-environmental behaviors (PEBs) can contribute to greener operations, but research on workplace PEBs is still an emerging field. This study examined how employees’ perceptions of environmental corporate social responsibility (CSR) policies and practices are related to their self-reported voluntary PEBs, including person-organization fit (P-O fit) and organizational identification’s role as sequential mediators. Data were gathered from 178 workers from different organizations via an online survey. The results reveal a positive relationship between perceived environmental CSR practices and work PEBs, which are both directly and indirectly connected through P-O fit and organizational identification. Managers can thus use environmental CSR activities to capitalize on employees’ P-O fit and organizational identification, thereby fostering work PEBS.

## 1. Introduction

Most business activities consume natural resources and/or emit pollutants and residuals as part of production processes. Companies have thus been held responsible by many for global warming and the environmental crisis. To address their societies’ demands for more sustainable development, organizations have been incorporating environmental concerns into their policies, namely, as part of corporate social responsibility (CSR) strategies [1,2]. CSR entails addressing social, environmental, and economic issues concurrently in business activities [3,4,5] in order to maximize the creation of shared value for human and non-human stakeholders while mitigating potential negative impacts [6,7,8].

In its environmental dimension, CSR includes context-specific policies and practices that eliminate and/or reduce companies’ negative impacts on the environment (e.g., less natural resource consumption and waste, clean energy, and green process-product innovations). CSR also contributes to environmental conservation (e.g., protection of endangered species and reforestation). Besides formalized pro-environmental practices selected by upper management, the literature suggests that workers’ pro-environmental behaviors (PEBs) can significantly contribute to improving organizations’ environmental performance [9,10,11,12]. 

PEBs consist of employees’ workplace behaviors that have a beneficial effect on the environment [13,14,15,16,17,18], such as turning off lights, saving water, separating materials for recycling, or advocating eco-friendly actions. Individually, PEBs may appear to be of little importance, but, as they spread throughout organizations, these behaviors can have a multiplier effect and contribute to the achievement of sustainability targets [10,19]. Given PEBs’ significant impact, various researchers have called for studies focusing on how environmental CSR practices influence workers’ PEBs [20,21,22]. The literature on work PEBs is still relatively new, so the process whereby organization-level factors, including environmental CSR practices, foster employees’ PEBs is still unclear [20,23,24]. 

**Figure 1:**
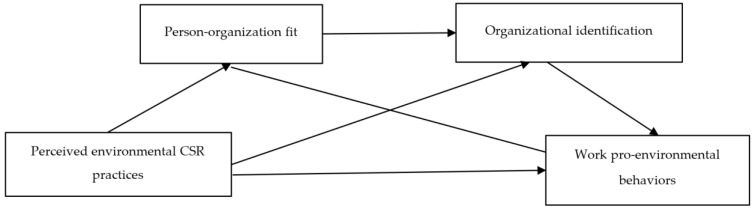
Conceptual model.

The present research sought to extend the current understanding of the psychosocial mechanisms underlying the relationship between workers’ PEBs and perceptions of environmental CSR practices. More specifically, this study examined person-organization fit (P-O fit) and organizational identification as potential sequential mediators. The theoretical model adopted (see Figure 1) proposes that employees’ perceived environmental CSR enhances their perceived P-O fit, which subsequently increases their organizational identification and thus work PEBs.

## 2. Research Background and Hypotheses Development

### 2.1. Environmental CSR and Work PEBs

Stakeholders currently expect organizations to behave in ethical and socially responsible ways that contribute to society’s welfare and imply being environmentally responsive and minimizing negative impacts on ecosystems. Past corporate environmental malpractices (e.g., abusive exploitation of forest areas leading to deforestation) and disasters (e.g., oceanic oil spills) have called the public’s attention to companies’ major adverse effects on the planet and its long-term sustainability. Although these corporate crises have greater visibility, organizations’ daily routines can also negatively affect the environment. 

Organizations have been encouraged by various institutions and pressure groups to adopt more sustainable practices and reduce their business models’ carbon footprint. Governments and supranational institutions around the globe have formulated legislation and soft law instruments to regulate and guide companies’ environmental performance (e.g., the European Commission’s European Green Deal launched in December 2020). Besides abiding by the relevant legislation, organizations can also define and implement non-mandatory environmentally friendly policies and practices to improve their operations’ environmental efficiency and actively contribute to enhancing the environment. These strategies can encourage staff members to adopt more proactive workplace attitudes and behaviors, including PEBs [15,20,21,24]. 

PEBs fit into two categories—in-role and extra-role—according to the literature on organizational citizenship behavior (OCB) [14,15,19,25]. While in-role performance is required and rewarded as part of workers’ regular duties, extra-role actions exceed existing job expectations, so these initiatives are discretionary and informal. Both types of behaviors contribute to organizations’ good functioning and business goal attainment. 

The present study focused on voluntary PEBs because, although these extra-role behaviors are not systematically monitored or rewarded, they can benefit organizations both directly by conserving energy and resources and indirectly by safeguarding the natural environment. Overall, PEBs ultimately contribute to more sustainable production [26]. Despite these OCBs’ potential contribution to organizational performance, they have received limited attention from researchers [23]. 

In response to market and societal demands, initiatives to promote PEBs have increased within organizations [27,28]. Individuals vary in the extent to which they perceive and value CSR activities but working for organizations committed to environmental causes has a positive effect on work PEBs [21,29]. Prior studies have confirmed that, the clearer employees’ perceptions of environmental CSR practices are, the stronger these workers’ engagement in work PEBs becomes. For instance, Raineri and Paillé [30] report that, when employees observe that their organization participates in eco-friendly programs, these individuals also assert that they have adopted more PEBs. Other researchers have published similar findings [20,21,22,24,31]. The following research hypothesis was thus defined for the current study:

**Hypothesis** **1** **(H1).** *Perceived environmental CSR practices are positively related to workers’ voluntary PEBs.*

### 2.2. Environmental CSR, P-O Fit, and PEBs

Corporate practices can influence individuals’ attitudes and behaviors directly and indirectly via specific socio-psychological processes. One important mechanism is P-O fit, or perceived compatibility between workers and their organization, which occurs when at least one party provides what the other requires or they share similar fundamental characteristics—or both [32]. Since CSR policies and practices indicate organizational values and attributes, employees’ perceptions of these aspects serve as input or a basis for their judgements about P-O fit. As a result, a positive relationship between perceived CSR and perceived P-O fit can be expected [33,34]. 

The literature on organizational behavior has established P-O fit as an important determining factor of individuals’ workplace attitudes and behaviors. Prior research has found evidence that workers who share their organization’s values and attributes are more committed to that organization, more satisfied with their work situation, and less prone to voluntarily leaving the organization. These employees also exhibit more prosocial behavior, amongst other desirable outcomes [35,36,37,38]. 

Similarly, stronger perceived compatibility between workers and their organization is most probably related to a deeper engagement in PEBs at work. This connection was recently confirmed by Mi et al. [39], who found that employees’ perceived P-O fit significantly predicts reported levels of in-role and extra-role green behaviors at work. Cheema et al. [33] assert that perceived fit can lead workers to want to support organizational CSR initiatives by adopting workplace PEBs. Given the above findings, the following hypothesis was generated for the present research:

**Hypothesis** **2** **(H2).** *The relationship between perceived environmental CSR and employees’ voluntary PEBs is mediated by perceived P-O fit.*

### 2.3. Environmental CSR, Organizational Identification, and PEBs

Organizational identification may also be an important mediator of the link between perceived environmental CSR practices and workers’ PEBs. The former construct is rooted in social identity theory [40] and defined as a perception of belongingness to an organization that leads individuals to define themselves in terms of their membership in that organization [41]. The level of employees’ identification with their organization is an important predictor of their attitudes and behaviors at work [42,43], with individuals engaging more often in behaviors that help their organization achieve its goals when they feel a strong identification with that entity [44]. Workers who more closely identify with their organization accept and internalize that company’s values, beliefs, and goals. As a result, these employees are motivated to engage in behaviors consistent with those ideals, principles, and objectives, which in turn benefits their organization [45,46].

Empirical research has confirmed that CSR is an important driver of workers’ organizational identification [22,45,47,48,49,50]. Working for a socially and environmentally responsible organization enables individuals to develop feelings of pride, self-respect, and distinctiveness that contribute to increased self-esteem and self-worth [45,48] and a closer identification with that organization. The latter, in turn, predisposes employees to engage in behaviors contributing to the attainment of collective goals such as environmental efficiency. 

Workers with stronger organizational identification are thus more likely to exhibit PEBs in the workplace [51]. The extant research provides support for the assumption that organizational identification mediates the relationship between employees’ perceived CSR and their PEBs [24,33,50]. The third research hypothesis defined for the current study was as follows:

**Hypothesis** **3** **(H3).** *The relationship between workers’ perceived environmental CSR and voluntary PEBs is mediated by organizational identification.*

### 2.4. Environmental CSR, P-O Fit, Organizational Identification, and PEBs 

This research’s theoretical model proposes that environmental CSR practices can boost employees’ voluntary PEBs by enhancing these individuals’ perceived P-O fit, thereby increasing their organizational identification. Previous research has documented CSR’s connection with P-O fit [33,34] and organizational identification [22,45,47,48,49,50]. However, the existing literature does not appear to include any studies that have examined perceived P-O fit and organizational identification’s sequential mediation in the relationship between environmental CSR practices and workers’ voluntary PEBs. The present research was, therefore, the first to explore how environmental CSR influences employees’ voluntary PEBs directly and indirectly through two sequential mediators: perceived P-O fit and organizational identification. 

The literature reviewed for this study reveals that environmental CSR policies and practices can increase workers’ perceived P-O fit by clarifying their organization’s values, attributes, and goals [33,34]. Employees’ perception of similarity or compatibility with their company can contribute to increased feelings of belongingness, making that organization a significant referent regarding self-definition [35,52]. Workers who identify strongly with their organization accept and internalize their employer’s ideals, beliefs, and objectives, so these individuals are motivated to engage in behaviors more consistent with that organization’s values [35,45,46]. These employees will thus probably adopt more voluntary PEBs at work [33,50]. 

The current study’s theoretical model proposes that workers’ perceptions of their company’s environmental CSR practices can influence these individuals’ PEBs through P-O fit and organizational identification, which function as intermediate elements in this socio-psychological process. Employees’ perceptions of their organization’s environmental policies, which indicate its values, attributes, and goals, increase these workers’ perceptions of how well their personal traits fit with the organization’s characteristics. Perceived P-O fit, in turn, helps staff members develop a stronger identification with their company. These employees then look for ways to contribute to the organization’s environmental targets, including adopting more voluntary work PEBs. To take the above findings into account, the final hypothesis formulated was as follows:

**Hypothesis** **4** **(H4).** *The relationship between workers’ perceived environmental CSR and voluntary PEBs is serially mediated by perceived P-O fit and organizational identification.*

## 3. Materials and Methods

### 3.1. Procedures and Participants

The data were collected via an online survey distributed to workers in order to conduct a correlational study guided by the research hypotheses. Using web browser-based methods to collect data for organizational research has long been recognized as a useful strategy [53]. The present research drew on a sampling frame of employees from different organizations to understand more fully how environmental CSR affects workers’ PEBs at work. The survey was made available on various social media platforms (Facebook, LinkedIn, and Twitter) and disseminated within the researchers’ social networks to recruit as many participants as possible. To assure that the participants had had sufficient contact with their organization’s policies and practices, a minimum of six months of tenure in the respondents’ current organization was set as an inclusion criterion. 

The survey was organized into three sections: informed consent (following the ethical standards guidelines of Portugal’s Order of Psychologists), research variables measures, and the workers’ socio-professional characteristics. Participation in the study was voluntary and anonymous, and all data were kept confidential. The instructions explained how to complete the survey to reduce the possibility of errors, and the absence of right or wrong answers was explicitly highlighted to diminish evaluation apprehension [54]. After the respondents gave their informed consent, they were asked to report their perceptions of their organization’s environmental CSR practices and of their own P-O fit, organizational identification, and PEBs. The last section contained items covering socio-professional characteristics (e.g., age, gender, education, tenure in the organization, business sector, and organization size).

Initially, 259 surveys were received. After the elimination of incomplete surveys and responses from respondents who did not meet the inclusion criterion, a total of 178 usable surveys were obtained, which comprised the non-probability convenience sample of this study. The G*Power software was used to calculate the sample size based on statistical power [55] and to certify the collected sample’s adequacy. A sample size of 138 was recommended to achieve a statistical power of 0.95 in the model testing phase. Since the present study’s sample size exceeded this number, it was deemed sufficiently large enough to test the model. The respondents had a mean age of 37.30 years (standard deviation [SD] = 10.56, minimum = 21 years; maximum = 66 years), and 59.0% were female. Most respondents had a higher education degree (70.8%), 23.6% had between 10 and 12 years of schooling, and 5.6% had completed 9 years of schooling or less. 

The workers surveyed had a mean tenure of 9.95 years in their current organization (*SD* = 9.51 years; maximum = 40 years), with 75.3% having a permanent employment contract and the remaining a fixed-term contract. Around one-fifth had a management position (20.2%). The respondents worked in business areas such as commercial services (11.9%), information and communication technologies (11.9%), public administration (10.2%), health and social services (10.2%), education (8.5%), and consultancy (8.5%). Around three-quarters of the participants worked for a private organization (75.1%) with for-profit business goals (71.2%). Slightly more than a half of the respondents (53.7%) worked for a small and medium-sized organization (i.e., with less than 250 employees), 13.3% for organizations with 250–500 employees, and 32.8% for organizations with more than 500 employees. 

Almost half of the workers surveyed said that their organization had pro-environmental policies (45.8%), while 19.2% said that their company did not have any such policies and 35.0% indicated that they did not know. Regarding environmental certification (e.g., ISO14000), only 26.6% of the respondents answered in the affirmative. A further 20.9% asserted that their organization did not possess any certification, and more than half of the participants did not know whether their corporation had any environmental certification (52.5%). 

### 3.2. Variables and Measures

The respondents indicated their level of agreement with each item on a 5-point Likert scale, except for the PEBs measure. On this scale, 1 means “Totally disagree,” while 5 means “Totally agree.” 

#### 3.2.1. Environmental CSR Practices (Predictor Variable)

Six items based on Duarte [56] and Turker’s [57] research measured the respondents’ perceptions of their organization’s environmental CSR practices. These items were as follows: 

“My organization makes an effort to reduce its impact on the environment.” 

“…makes an effort to reduce the natural resources used during its functions (e.g., water and energy).” 

“…separates materials and waste for recycling.” 

“…develops projects aiming to protect the natural environment.” 

“…gives donations to associations that protect the environment.” 

“…encourages its employees to participate in voluntary activities designed to protect the environment.” 

A composite score for each participant was calculated by averaging the set of items’ scores (alpha [*α*] = 0.85). Higher values represent perceptions of stronger environmental CSR. 

#### 3.2.2. P-O Fit (Mediator Variable One)

The participants’ perceptions of the fit between their personal characteristics and values and those of their organization were measured using three items adapted from Cable and Judge’s [58] research. These were “this organization’s values and ‘personality’ reflect my own values and personality,” “my values match those of current employees in the organization,” and “my values are compatible with and adjusted to this organization.” Each respondent’s composite score was calculated by averaging the three items (*α* = 0.84). Higher scores represent a better perceived P-O fit.

#### 3.2.3. Organizational Identification (Mediator Variable Two)

The respondents’ identification with their organization was assessed using four items from Mael and Ashforth’s [41] scale. These were, “when someone criticizes this organization, it feels like a personal insult”; “if a story in the media criticized this organization, I would feel embarrassed”; “when I talk about this organization, I usually say ‘we’ rather than ‘they’ ”; and “I am very interested in what others think about this company.” Each participant’s composite score was estimated by averaging this set of items (*α* = 0.76). Higher scores represent stronger identification with the relevant organization.

#### 3.2.4. Work PEBs (Criterion Variable)

The respondents’ workplace PEBs were measured using seven items based on Greaves et al. [59], Mouro and Castro [60], and Robertson and Barling’s [16] research: 

“I advocate for the importance of engaging in environmentally friendly behaviors.” 

“…offer to participate in environmental protection initiatives promoted by my organization.” 

“…make suggestions about how my organization can become more environmentally friendly.” 

“…turn off the lights when I leave a room.” 

“…use as little water as possible.” 

“…shut down equipment after using it.” 

“…separate materials and waste for recycling.” 

The workers surveyed indicated how frequently they performed each behavior in their workplace on a 5-point Likert scale (1 = “Never”; 5 = “Very frequently”). A composite score for each participant was calculated by averaging all the items’ score (*α* = 0.69). Higher values represent more work PEBs.

### 3.3. Common Method Variance (CMV)

Given that the data for each construct were obtained from the same source at a single moment in time, CMV could threaten the results’ validity. As suggested by Podsakoff et al. [54] different rating scales were used, the respondents’ anonymity and their answers’ confidentiality were guaranteed, and the absence of right or wrong answers was emphasized in the instructions to prevent CMV [53]. In addition, Harman’s single factor test was conducted by carrying out unrotated exploratory factor analysis with the 20 items included in the survey. The results show that the first factor explains less than 50% (i.e., 28.54%) of a total of the 63.01% of all variance explained by the model’s variables (Kaiser-Meyer-Olkin = 0.81; *p* < 0.001), which suggests that CMV does not seriously weaken the results’ validity.

## 4. Results

Statistical analyses were conducted using IBM SPSS Statistics version 26 software, and sequential mediation analysis was done with the PROCESS macro program [61]. Table 1 presents the means, SDs, and Spearman’s correlation coefficients. The selected variables are all positively and significantly correlated with each other, as shown by their low to moderate correlation coefficients. 

The respondents’ tenure in their organization and attainment of a management position are positively and significantly correlated with their PEBs (*rho* = 0.22 and *rho* = 0.24, respectively; both *p* < 0.01). The participants’ age also has a positive relationship with their PEBs (*rho* = 0.32; *p* < 0.01). Since these workers’ age and tenure were strongly correlated (*rho* = 0.75; *p* < 0.01), the decision was made to include only tenure and attainment of a management position as covariates in subsequent analyses. 

The PROCESS macro was used to evaluate the mediation effects because it is widely used in social and business sciences and is an easy-to-use tool for estimating direct and indirect effects in multiple mediator models. The variance inflation factor (VIF) and tolerance values were checked to rule out the possibility of multicollinearity. The VIF values range from 1.25 and 1.65 and tolerance values between 0.61 and 0.80, which effectively addresses any concerns about multicollinearity [62]. Table 2 presents the results obtained using the software’s Model 6 in the sequential mediation analysis.

The first hypothesis (i.e., H1) proposes that a positive relationship exists between workers’ perceptions of their organization’s environmental CSR practices and their voluntary work PEBs. As shown in Table 2, perceived environmental CSR’s effect on PEBs is statistically significant (non-standardized coefficient [*B*] = 0.15; *p* < 0.01), indicating that organizations’ adoption of environmentally responsible practices increases their employees’ workplace PEBs. H1 is, therefore, supported by the results. 

The second hypothesis (i.e., H2) states that workers’ P-O fit mediates the path between their perceived environmental CSR and work PEBs. The results confirm that employees’ perceptions of environmental CSR practices significantly predict these individuals’ opinions about the similarity between their own values and characteristics and those of their organization (*B* = 0.46; *p* < 0.001). However, P-O fit does not significantly predict the reported levels of workplace PEBs (*B* = −0.02; non-significant [n.s.]), so this factor’s indirect effect is negligible (*B* = −0.01; 95% confidence interval (*CI*) = −0.06; 0.05). H2 is thus not supported by the results. 

The third hypothesis (i.e., H3) posits that organizational identification also has a mediating effect on the relationship between workers’ perceptions of their organization’s environmental CSR practices and these individuals’ voluntary work PEBs. The results indicate that, although organizational identification helps explain employees’ PEBs (*B* = 0.17; *p* < 0.05), their perceived environmental CSR does not have a significant impact on how much workers identify with their employer (*B* = 0.10; *n.s*.). This variable’s indirect effect is not statistically significant (*B* = 0.02; 95% *CI* = −0.01; 0.05), thereby negating that a significant mediating effect exists. H3, therefore, lacks empirical support.

**Figure 2:**
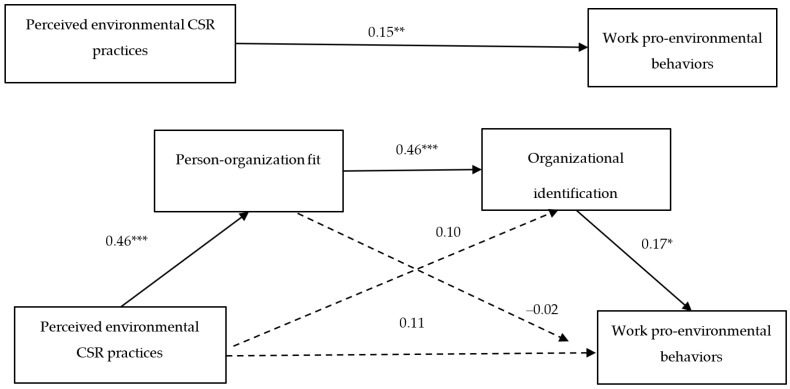
Perceived environmental corporate social responsibility practices’ effect on work pro-environmental behaviors through person-organization fit and organizational identification. (Notes: The values are non-standardized coefficients; * *p* < 0.05; ** *p* < 0.01; *** *p* < 0.001; participants’ tenure in organization and managerial position entered as covariates; CSR = Corporate social responsibility).

The last hypothesis (i.e., H4) states that employees’ P-O fit and organizational identification serially mediate the relationship between their perceived environmental CSR and voluntary work PEBs. The results confirm that perceived environmental CSR has a significant indirect effect on PEBs through the mediation of P-O fit and organizational identification (*B* = 0.03; 95% *CI* = 0.01; 0.07). Therefore, workers’ perceptions of their organization’s adoption of environmentally responsible practices are associated with these individuals’ perceived better P-O fit (*B* = 0.46; *p* < 0.001). This connection in turn fosters higher levels of identification with their organization (*B* = 0.46; *p* < 0.001), which is then associated with more workplace PEBs (*B* = 0.17; *p* < 0.05). These findings provide support for H4. The final version of the research model explains 18% of the unique variance of voluntary PEBs [*F*(5,172) = 7.74; *p* < 0.001]. Figure 2 presents this study’s main results.

## 5. Discussion and Conclusions

Drawing on P-O fit and social identity theories [32,40,41], the present empirical research tested a sequential pathway model explaining how employees’ perceptions of their organization’s environmental CSR practices foster these workers’ voluntary PEBs on the job. This study sought to expand the existing literature on these behaviors by offering new insights into the ways organizations can foster PEBs among their staff members [17,20,21,22,23,31,63].

The current findings include a positive association between workers’ perceived environmental CSR and voluntary PEBs, which confirms H1. Other authors have reported similar results [20,21,22,24,30], so the present study fits into this body of research, providing further evidence that a positive relationship exists between the two constructs. Corporate environmental practices promote their employees’ adoption of environmentally friendly behaviors so that the more workers see their company as committed to environmental causes, the more likely these individuals are to engage in voluntary PEBs at work.

H2 proposed that P-O fit has a mediating effect on the relationship between perceived environmental CSR practices and voluntary work PEBs, but the results provide no empirical support for this hypothesis. In line with what was expected, perceived environmental CSR practices and P-O fit have a significant connection, which indicates that organizations’ practices signal their values and attributes and contribute to their workers’ perceived P-O fit [33,34]. However, employees’ subjective P-O fit does not significantly predict their greater engagement in workplace PEBs, which inhibits the expected mediating effect. Previous studies have found that workers’ P-O fit perceptions significantly predict self-reported in-role and extra-role green behaviors at work [39] and that perceived P-O fit explains the process whereby companies’ activities promote employees’ work PEBs [33]. Additional studies are thus needed to clarify these mechanisms further.

Unexpectedly, the current results indicate that organizational identification has no statistically significant mediating effect on the relationship between perceived environmental CSR practices and voluntary workplace PEBs, so H3 was rejected. Organizational identification meets one of the conditions for mediation (i.e., explains the adoption of PEBs), but perceived environmental CSR apparently does not explain respondents’ levels of identification with their employer, thereby weakening any significant mediation effect. This finding is contrary to prior studies, which have found that CSR practices foster workers’ organizational identification [22,45,47,48,49,50] and that the latter mediates the relationship between workers’ perceived CSR and their PEBs [24,33,50]. A possible explanation for the present research’s lack of support for H2 and H3 could be the intervention of moderating variables not considered in this study, such as attitudinal ambivalence, which can be a potential barrier to workers’ adoption of PEBs on the job [64,65]. Future studies must reexamine these findings to confirm their stability across research contexts.

Finally, H4 is supported by the results. This study thus found evidence that workers’ P-O fit and organizational identification are sequential mediating psychosocial mechanisms explaining the relationship between these individuals’ perceived environmental CSR practices and voluntary work PEBs. This finding adds new information to the existing knowledge about the relationship between the four variables. Although prior research has examined some of the relationships between these variables [22,33,34,45,47,48,49,50], no clear evidence was found of existing studies that examined employees’ perceived P-O fit and organizational identification’s role as sequential mediators in the relationship between environmental CSR practices and voluntary workplace PEBs.

The current results support the conclusion that significant indirect relationships exist between the research model’s two main variables via the proposed mediating variables. As suggested in the theoretical background section, this connection may be present because environmental CSR policies and practices signal organizations’ values, attributes, and goals, which increases workers’ perceived P-O fit [33,34] and leads to stronger feelings of belongingness and identification with their organization [52]. This deeper identification subsequently motivates employees to take part in behaviors consistent with their company’s ideals, characteristics, and objectives [35,45,46] and to adopt more voluntary work PEBs [24,33,50]. While the extant research has examined each variable’s mediating role separately, the present findings add to the literature by highlighting their combined and sequential explanatory effects.

### 5.1. Limitations and Suggested Future Research

Despite the interesting results above, various limitations need to be considered when interpreting the findings and defining avenues of further research. First, this study’s cross-sectional design restricts any inferences regarding causal relationships between the variables. Although the proposed model reflects theoretical tenets and previous research findings, the empirical robustness of the evidence for these connections should be confirmed by longitudinal research.

A second limitation is the danger of common method bias due to the cross-sectional data being collected from a single source [54]. This bias was minimized by following recommended procedures while developing the questionnaire, including guaranteeing the data’s anonymity [54]. In addition, exploratory factor analysis confirmed that the present results’ validity was not severely weakened by the adopted method. Future research, nonetheless, needs to test the proposed mediating effects further using time-lagged data collection.

Third, only self-reported PEBs were measured, which may not be a fully reliable indicator of actual PEBs [66]. The respondents could have been motivated to present their behavior in a favorable light, and pro-environmental concerns and actions have a normative weight that can strengthen this motivation to exaggerate. Future studies could collect data on workers’ PEBs from other sources (e.g., direct supervisors or close colleagues) or use more objective measures (e.g., kilograms of separate garbage).

Finally, the data were gathered through social networks from a non-probabilistic sample, which may limit the generalizability of the results. Convenience sampling can contribute to the recruitment of respondents who are more strongly disposed to participating in this type of research and who feel more comfortable using the Internet and online tools. However, the demographic profile of the surveyed workers indicates that the sample varied in both age and type and size of organization, which indicates that this issue is not of major concern. Further studies are needed, however, to re-test the proposed model using probability sampling techniques. Data collection using paper-and-pencil surveys may help to gain access to respondents less familiar with online tools.

Despite these limitations, the findings contribute useful insights to the literature and open new avenues of research. Future investigations could focus on clarifying whether environmental CSR’s impact on PEBs is constrained by employees’ perceptions of their organization’s other motivations (e.g., profit and reputation) for endorsing pro-environmental activities besides protecting the environment [21]. Previous studies have also shown that CSR has a strong impact on workers with moderate biospheric values, which suggests that CSR works as a contextual cue for those who are already concerned about the environment, thereby reinforcing workplace PEBs [21]. Another related line of research would be to examine if biospheric values moderate the relationships between environmental CSR and the proposed model’s remaining variables.

Organizational identification has been studied mainly with regard to its positive outcomes, including extra-role efforts [42,43], but its influence on voluntary PEBs has received insufficient attention. Organizational identification might not have the same effect on every one of these behaviors, or other mechanisms could intervene in its impact on this type of OCB. For instance, if workers feel ambivalent toward their organization, then the link between organizational identification and PEBs can be weakened [67]. Researchers could explore how organizational identification affects the adoption of specific PEBs (e.g., car sharing for home-work commutes and energy efficiency) and whether identification with some aspects of organizations—but not others—may create barriers to these behaviors.

### 5.2. Theoretical and Practical Implications

The present results have various theoretical and practical implications. This research responded to calls for studies of facilitators of workplace PEBs [16] and confirmed that environmental CSR is a significant predictor of employees’ PEBs. By providing a more complex perspective on corporate environmental policies and practices’ impacts, the current findings stress the need to consider P-O fit and organizational identification simultaneously when exploring the dynamics of voluntary workplace PEBs. The sequential effects of these two variables had not been previously explored, and the results confirm the importance of examining how multiple mechanisms contribute to greater engagement in voluntary PEBs. The findings thus complement the extant research and extend it by providing a more complete understanding of the mechanisms driving voluntary work PEBs.

At a practical level, the results indicate that managers can encourage their workers’ PEBs by making their organization’s pro-environmental objectives and activities more explicit to their employees. This strategy implies clearer communication about environmental policies and practices. Stakeholders’ low awareness of organizations’ CSR activities has been identified as a critical impediment to maximizing these initiatives’ business benefits [68]. In the present study, nearly half of the respondents were unsure whether their organization had environmental policies or certifications. Communication about organizations’ activities could thus be improved in these corporate settings.

Providing suitable training programs about environmental issues and making infrastructure available in company facilities (e.g., for waste separation) can also be an important way to strengthen workers’ perceptions of their organization’s commitment to protecting the environment. Perceived environmental CSR can, in turn, encourage employees to adopt more PEB. Managers’ personal engagement in and support of environmentally friendly activities is crucial as these individuals serve as ambassadors of corporate values who can inspire their workers to become more proactive [9,19].

Companies’ CSR activities appear to have a significant positive impact on their workers’ clearer perceptions that their organization has environmental goals and concerns aligned with their personal values. The perceived fit between workers and their organization then fuels a stronger identification with their employer and, in turn, engagement in more workplace PEBs. Managers who seek to foster higher levels of perceived P-O fit and organizational identification can implement and promote CSR initiatives linking organizational and personal goals and values.

In addition to improving PEBs on the job, these two factors contribute to many other desirable attitudes and behaviors at work, including better individual performance and a lower intention to quit [35,36,37,38,42,43]. Managers can thus use environmental CSR activities to capitalize on employees’ P-O fit and organizational identification, thereby fostering work PEBS and, concurrently, their company’s environmental efficiency. This strategy may also provide more generalized collateral benefits and stimulate better overall performance and efficiency.

## Figures and Tables

**Table 1:** Means (M), standard deviations (SD), correlations, and Cronbach’s alphas ^1^.

Variables	M	SD	1	2	3	4	5	6
1. Tenure in organization	9.95	9.51	-					
2. Management position ^†^	-	-	0.26 **	-				
3. Environmental CSR	3.16	0.81	0.18 *	0.23 **	(0.85)			
4. Person-organization fit	3.26	0.92	0.14	0.13	0.41 **	(0.84)		
5. Organizational identification	3.48	0.81	0.12	0.26 **	0.37 **	0.57 **	(0.76)	
6. Work PEBs	3.70	0.63	0.22 **	0.24 **	0.28 **	0.19 *	0.31 **	(0.69)

Notes: ^†^ management position: 0 = no, 1 = yes; * *p* < 0.05; ** *p* < 0.01; CSR = corporation social responsibility; Cronbach’s alphas in parentheses; PEBs = pro-environmental behaviors.

**Table 2:** Regression coefficients (B), standard errors (SEs), model summary information, and indirect effects for the sequential mediation model ^1^.

Variables	P-O Fit (Mediator 1)		OI (Mediator 2)		PEBs (Criterion Variable)	
*Total effects*	*B*	SE	*B*	SE	*B*	SE
Constant	-	-	-	-	3.05 ***	0.18
Perceived environmental CSR Practices (ECSR)	-	-	-	-	0.15 **	0.06
Tenure in organization	-	-	-	-	0.01 **	0.01
Management position ^†^	-	-	-	-	0.26 *	0.00
					*F*(3,174) = 10.24; *p* < 0.001; *R*^2^ = 0.15	
*Direct effects*						
Constant	1.75 ***	0.26	1.56 ***	0.22	2.69 ***	0.22
ECSR	0.46 ***	0.08	0.10	0.07	0.11	0.06
P-O fit	-	-	0.46 ***	0.06	−0.02	0.06
OI	-	-	-	-	0.17 *	0.07
Tenure in organization	0.01	0.01	0.00	0.01	0.01 **	0.01
Management position	0.07	0.16	0.35 **	0.13	0.20	0.12
	*F*(3,174) = 12.91; *p* < 0.001; *R*^2^ = 0.18		*F*(4,173) = 28.21; *p* < 0.001; *R*^2^ = 0.40		*F*(5,172) = 7.74; *p* < 0.001; *R*^2^ = 0.18	
*Indirect effects*	Effect (BootSE)		BootLLCI		BootULCI	
Total	0.04 (0.03)		−0.01		0.11	
ECSR→P-O fit→PEBs	−0.01 (0.03)		−0.06		0.05	
ECSR→OI→PEBs	0.02 (0.01)		−0.01		0.05	
ECSR→P-O fit→OI→PEBs	0.03 (0.02)		0.01		0.07	

Notes: CSR = corporate social responsibility; P-O = person-organization; OI = organizational identification; PEBs = pro-environmental behaviors; * *p* < 0.05; ** *p* < 0.01; *** *p* < 0.001; ^†^ management position: 0 = no, 1 = yes; LLCI = lower limit confidence interval; ULCI = upper limit confidence interval.

## Data Availability

The data will be made available on reasonable request by contacting the corresponding author.
